# Influence of PCL and PHBV on PLLA Thermal and Mechanical Properties in Binary and Ternary Polymer Blends

**DOI:** 10.3390/molecules27217633

**Published:** 2022-11-07

**Authors:** Raasti Naseem, Giorgia Montalbano, Matthew J. German, Ana M. Ferreira, Piergiorgio Gentile, Kenneth Dalgarno

**Affiliations:** 1 School of Engineering, Newcastle University, Newcastle upon Tyne NE1 7RU, UK; 2 Department of Applied Science and Technology, Politecnico di Torino, 10129 Torino, Italy; 3 School of Dental Sciences, Translational and Clinical Research Institute, Faculty of Medical Sciences, Newcastle University, Newcastle upon Tyne NE1 7RU, UK

**Keywords:** polymer blends, polyester biomaterials, PLLA, PCL, PHBV

## Abstract

PLLA, PCL and PHBV are aliphatic polyesters which have been researched and used in a wide range of medical devices, and all three have advantages and disadvantages for specific applications. Blending of these materials is an attractive way to make a material which overcomes the limitations of the individual polymers. Both PCL and PHBV have been evaluated in polymer blends with PLLA in order to provide enhanced properties for specific applications. This paper explores the use of PCL and PHBV together with PLLA in ternary blends with assessment of the thermal, mechanical and processing properties of the resultant polymer blends, with the aim of producing new biomaterials for orthopaedic applications. DSC characterisation is used to demonstrate that the materials can be effectively blended. Blending PCL and PHBV in concentrations of 5–10% with PLLA produces materials with average modulus improved by up to 25%, average strength improved by up to 50% and average elongation at break improved by 4000%, depending on the concentrations of each polymer used. PHBV impacts most on the modulus and strength of the blends, whilst PCL has a greater impact on creep behaviour and viscosity. Blending PCL and PHBV with PLLA offers an effective approach to the development of new polyester-based biomaterials with combinations of mechanical properties which cannot be provided by any of the materials individually.

## 1. Introduction

Orthopaedic procedures are seemingly ever increasing due to both an increase in the aging population and a rise in obesity rates, contributing to the increase in bone fractures. Permanent implants are expected to serve for the whole term of a patient’s life, in contrast to temporary implants, which are required for a shorter time period to allow for the healing of broken bones [1]. Current orthopaedic implants of both categories are produced from metallic materials, which display adequate mechanical properties and fatigue and corrosion resistance; however, the stiffness of metallic materials is greater than that of bone, and this can cause stress shielding [2,3]. As a less stiff alternative to metal implants, aliphatic polyesters are a group of biocompatible and bioresorbable polymers used in a wide range of biomedical applications. Some of the most commonly studied polymers include polylactide (PLA), polycaprolactone (PCL) and polyhydroxyalkanoates (PHA) [4,5,6].

Each of these polymers has advantages and disadvantages for biomedical applications [7]. Poly l-lactide (PLLA) has been used in a range of medical devices [8], including various orthopaedic clinical applications [9], where its favourable mechanical properties when compared to other biopolymers offers a performance advantage. PLLA has a high tensile strength, low ductility and is a semi-crystalline polymer. The crystalline structure can make PLLA mechanically superior when compared to other polyesters in load-bearing applications [4,9,10,11]. PLLA has glass transition and melting temperatures of 55–80 °C and 170–180 °C, respectively. In contrast, polycaprolactone (PCL) is a soft, semi-crystalline polymer with a low melting and glass transition temperatures (55–60 °C and −54 °C, respectively) [12,13]. Although it possesses high ductility (with a tensile elongation at break of over 700%) and a high impact strength, it has a low tensile strength (~23 MPa) and Young’s modulus [3,5,14,15,16]. Poly(3-hydroxybutyrate-co-3-hydroxyvalerate) (PHBV) is an aliphatic biodegradable polyester with a melting temperature between 80 and 160 °C and a glass transition temperature in the range of −5 to 20 °C depending on the HV (hydroxyvalerate) content of the copolymer, which can be adjusted to control the mechanical properties and degradation of the polymer [17]. PHBV has been shown to produce consistent favourable bone tissue adaptation response in addition to the elimination of any undesirable chronic inflammatory responses (up to 12 months after implantation) [18].

Both PLLA and PHBV are hard polymers with poor impact performance [4,5,9,19], which limits their use. The brittle nature of the polymers can be improved through blending with soft ductile polymers [12]. Blending of polymers is a simple yet effective method to obtain new materials with enhanced properties, as the limitations of the dominant component in the blend can be mitigated. Tuning of the physical and mechanical properties of a blend can be achieved with the selection of appropriate materials, adjustment of the blend compositions and appropriate preparation conditions [14]. The blending of PLA/PCL and PLA/PHBV as two co-polymer blends for use in biomedical applications has been previously investigated [6,20,21,22,23]. These studies have shown that it is possible to increase the fracture toughness or elongation at break of PLLA by blending with PCL or PHBV, but with reduced modulus or tensile strength [12,24,25,26]. Ternary blends of the three materials have not been previously assessed; however, the three materials together offer an attractive combination: PLLA providing strength, PCL giving ductility and PHBV enhancing biocompatibility of the material whilst also contributing to the mechanical properties [27].

Ternary blends with polymers which are not established biomaterials have previously been considered in terms of their ability to modify the behaviour of PLLA, PCL and/or PHBV using (i) polypropylene carbonate (PPC) in PLLA/PHBV/PPC blends [28], which showed reduced strength and modulus compared to PLLA alone; (ii) poly(butylene succinate) (PBS) in PLLA/PHBV/PBS blends [29], which showed reduced strength and modulus but increased elongation at break compared to PLLA alone; and (iii) montmorillonite (MMT) nanoclay in PLA/PCL/MMT-nanoclay blends [30], which increased modulus and strength compared to the PLLA/PCL blend, but reduced toughness and impact strength. These studies reinforce the observation from previous studies with PLLA/PCL and PLLA/PHBV blends that a formulation which provides increased ductility without sacrificing modulus and strength has yet to be found.

The aim of this study was, therefore, to characterise binary and ternary blends of PLLA, PCL and PHBV produced through twin screw extrusion for their suitability for use in orthopaedic applications in terms of mechanical performance and processability. Changes in microstructure are assessed via FTIR and DSC, with mechanical properties characterised in tension using modulus, strength, creep rate and viscosity as the key indicators of processability and mechanical behaviour over extended time periods. As orthopaedic devices are commonly applied for extended time periods, the creep behaviour of the blended materials was considered an important property to consider, and one which had not been studied in previous work on blended polyester formulations.

## 2. Materials and Methods

### 2.1. Materials

The base polymers were PLLA (PURASORB PL38, Corbion Purac, Amsterdam, The Netherlands), PHBV with 8% HV content (Sigma-Aldrich, St. Louis, MO, USA) and PCL (average Mn 80,000; Sigma-Aldrich). A range of two-component and three-component blends were evaluated using the three polymers detailed, using at least 70% PLLA, and at least 5% of either PHBV or PCL or both. Four specific blends were selected for in-depth study: PLLA/PHBV 85:15; PLLA/PCL 70:30; PLLA/PCL/PHBV 90:5:5 and 80:10:10.

### 2.2. Extrusion

**Figure 1:**
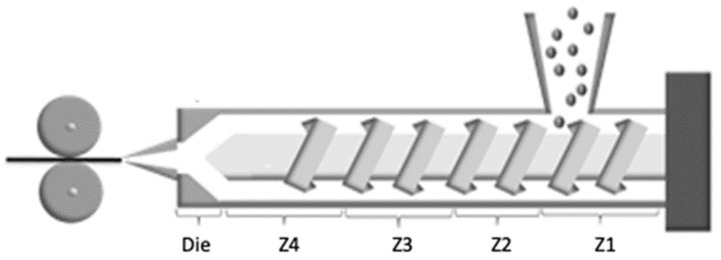
Schematic of extruder with temperature zones.

Filament was extruded in batches of 20 g of polymer. A Microlab 10 mm twin screw melt extruder connected to a twin belt haul-off (Rondol, France) was used for filament production. Laser measurement at the end of the haul off machine allows for a live reading of filament diameter, which was nominally 1.75 mm. Figure 1 illustrates the locations of the 5 temperature zones along the extruder barrel, and Table 1 shows the extrusion temperatures in the zones for each material. Attempts to extrude PHBV alone were unsuccessful in producing usable filament due the high temperature sensitivity of the polymer and associated thermal degradation. Melt processing of PHBV is known to be difficult to achieve [31,32]. The extrusion temperatures used for extrusion of the blends were the same as for PLLA.

### 2.3. Mechanical Properties

A universal tester (AGS-X autograph, Shimadzu, Kyoto, Japan) was used to conduct mechanical tests on polymer filaments using a 1 kN load cell. All tests were conducted at 22 °C. Filaments were subjected to a 5 N pre-load, the gauge length was 300 mm and materials were tested in triplicate. Additionally, 30 mm/min was used for the displacement-based test and termination was at the point of material failure or 300 mm displacement. 

Creep tests were also carried out on filaments using the universal tester and a 1 kN load cell. A displacement loading rate of 10 mm/min was applied to the specimens until a load of 40 N was reached, then held for a duration of 3 h or until the sample reached a maximum displacement of 0.5 m. The initial gauge length was again 300 mm, with tests carried out in triplicate. The change in displacement for each polymer sample for the test duration was evaluated by calculating a steady state creep rate ($\dot{\varepsilon }$):

(1) $$\dot{\varepsilon }=\frac{\varepsilon_{8000}}{\varepsilon_{50}}$$

where *ɛ*_8000_ and *ɛ*_50_ were the strains measured after 8000 and 50 s, respectively.

### 2.4. Attenuated Total Reflectance—Fourier Transform Infrared (ATR-FTIR)

An FT-IR Spectrometer (Spectrum Two, Perkin Elmer) was used for chemical analysis. Spectra were recorded from 4000 to 500 cm^−1^ and 16 scans per specimen (spectral resolution 4 cm^−1^). All output data were baseline corrected and normalised using Spectrum Quant software (Perkin Elmer).

### 2.5. Differential Scanning Calorimetry (DSC)

Differential scanning calorimetry (DSC823e, Mettler Toledo) was used to assess the thermal profile of the materials. A heating rate of 10 °C/min was applied in the range of 25–240 °C. Aluminium crucibles were used as the reference and sample holder which contained a known weight of polymer prior to analysis. Material crystallinity was calculated using Equation (2) [33]:

(2) $$Crystallinity\;\left(\%\right)=\left(\;\frac{\Delta H_{m}-\Delta H_{cc}}{\;\Delta H_{m100\%}}\right)$$

where Δ*H_m_* is the melting enthalpy, Δ*H_cc_* is enthalpy of cold crystallisation and Δ*H_m_*_100%_ is the enthalpy of melting for 100% crystalline polymer being investigated. For PLLA, PCL and PHBV, Δ*H_m_*_100%_ is reported in the literature as 93.7 J/g, 139 J/g and 146.6 J/g, respectively [6,34,35]. For polymer blends, the crystallinity for each polymer in the blend was calculated according to the relative weight of that polymer in the blend and these quantities added together to give the total polymer crystallinity.

### 2.6. Rheological Assessment

Rheological analyses were conducted on three samples for each material using a DHR-2 controlled stress rotational rheometer (TA Instruments, New Castle, DE, USA) equipped with an environmental test chamber for temperature control during testing. Analysis was conducted using a 25 mm parallel plate geometry, keeping a constant temperature of 210 °C. Frequency sweep tests were performed to observe the variation in the storage modulus (G’) and loss modulus (G’’) with increasing angular frequency (from 1 to 600 rad/s). The values obtained from the frequency sweep analysis were used to obtain the material flow ramps by applying the Cox–Merz equation, where the variation in the material viscosity was obtained for increasing shear rates (1–600 s^−1^).

## 3. Results

### 3.1. Thermal and Chemical Analysis

**Figure 2:**
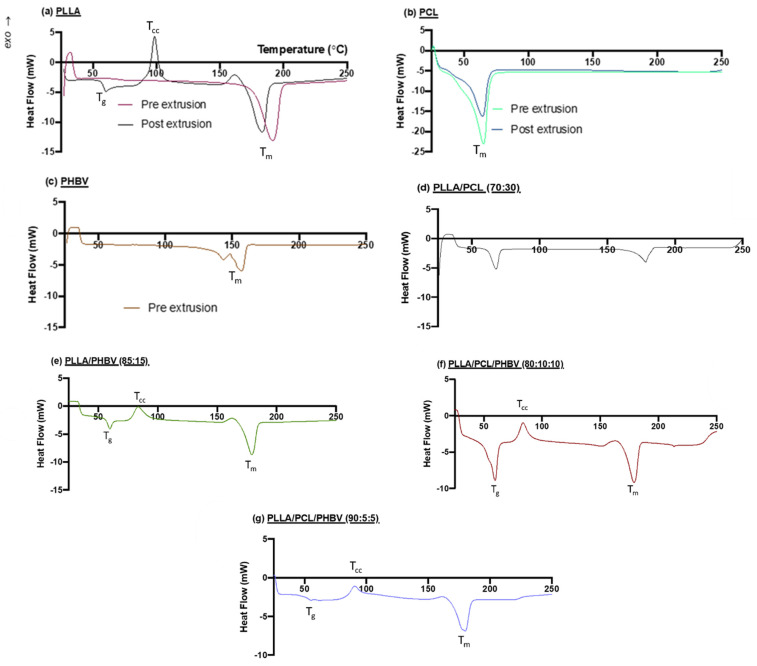
DSC curves (**a**) PLLA, (**b**) PCL, (**c**) PHBV, (**d**) PLLA/PCL (70:30), (**e**) PLLA/PHBV (85:15), (**f**) PLLA/PCL/PHBV (80:10:10) and (**g**) PLLA/PCL/PHBV (90:5:5).

DSC curves illustrating the thermal characteristics of the base polymers and blends are shown in Figure 2 and quantified in Table 2. For the base materials investigated pre- and post-extrusion (Figure 2a,b), there is a notable difference for PLLA and a subtle change for PCL post-extrusion in comparison to pre-extrusion. After extrusion, PLLA presents a crystallisation peak, along with lower T_g_ and T_m_ peaks. In contrast, the melting peak of PCL does not appear to be affected by the extrusion process. PHBV pre-extrusion shows two melt peaks (Figure 2c) which relate to the two constituents of the polymer, PHB and HV, with PHB representative of the higher melting peak. T_g_ and T_m_ remain close to that of PLLA in all blends, but the location of the peak is modified by parts of the blends becoming more mobile at lower temperatures.

The two-polymer blend profiles are illustrated in Figure 2d,e. Incorporation of 30% PCL in a blend with PLLA eliminates the crystallisation process for PLLA, whereas it remains present with PHBV incorporation. The step change glass transition for PLLA is accentuated with the incorporation of PCL (Figure 2d). Here, we see the fusion of the T_g_ of PLLA together with the T_m_ of PCL at 68 °C. For both two-polymer blends, the T_g_ and T_m_ are similar to those for PLLA post-extrusion. The three-polymer blend profiles are shown in Figure 2f,g, and there is a clear change in the glass transition and crystallisation peaks when more PCL/PHBV is incorporated into PLLA. The melting temperatures of the three-component blends remain in a 4 °C range of the PLLA post-extrusion T_m_.

Table 2 also details the melt enthalpy (Δ*H_m_*) values for the polymers and the cold crystallisation enthalpy (Δ*H_cc_*), which allows the degree of crystallisation to be estimated. The effect of thermal processing on PLLA and PCL can be seen from the decline in material crystallinity from pre to post-extrusion, with decreases of 49.4% and 8.9% for PLLA and PCL, respectively. PHBV has the lowest melt enthalpy of all the polymers. For PLLA/PCL (70:30), the melting peak at ~178 °C is reduced by PCL incorporation into PLLA, lowering the melt enthalpy by 26.39 J/g. The melt enthalpy for PLLA/PHBV (85:15) is not as significantly affected by PHBV incorporation (4.12 J/g enthalpy reduction). The material crystallinity of PLLA/PCL is impacted when the two polymers are combined; however, the crystallinity of the PLLA/PHBV (85:15) blend is improved when combining these two polymers.

**Figure 3:**
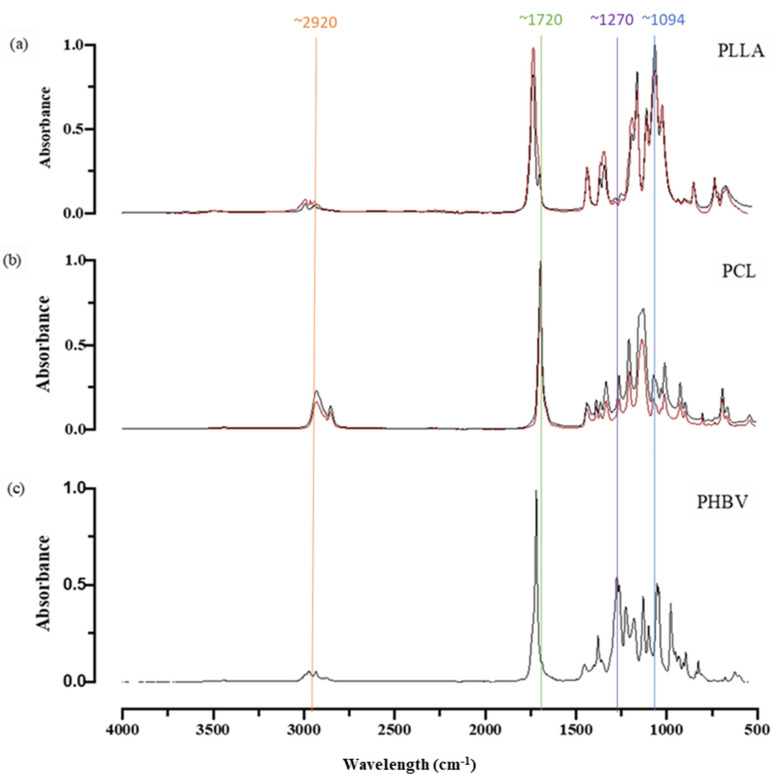
ATR-FTIR spectra for (**a**) PLLA, (**b**) PCL and (**c**) PHBV; the black lines are indicative of pre-extrusion and red lines of post-extrusion. Vertical lines indicate key wavelengths for ease of interpretation.

Figure 3 shows the ATR-FTIR spectra, which have been normalised and baseline corrected. All the spectra display similar profiles. Peaks at ~2920 and ~1720 cm^−1^ are characteristic peaks of C–H bonds and C=O carbonyl bonds, respectively. The peak located at ~1270 cm^−1^ represents the C–O saturated ester bonds [36]. PLLA shows an increase in the intensity of the peaks at ~1200 (C–C), ~1094 (C–O), ~1750 (C=O), ~1079–1189 (CH3) and ~1184/1088 (C–O–C) after extrusion [16,37,38,39]. With PCL, there is an increase in intensity of the fingerprint region (500–1500 cm^−1^) post-extrusion. PLLA, PCL and PHBV all have ATR-FTIR profiles that are indicative of them being aliphatic polyesters.

### 3.2. Mechanical Properties

**Figure 4:**
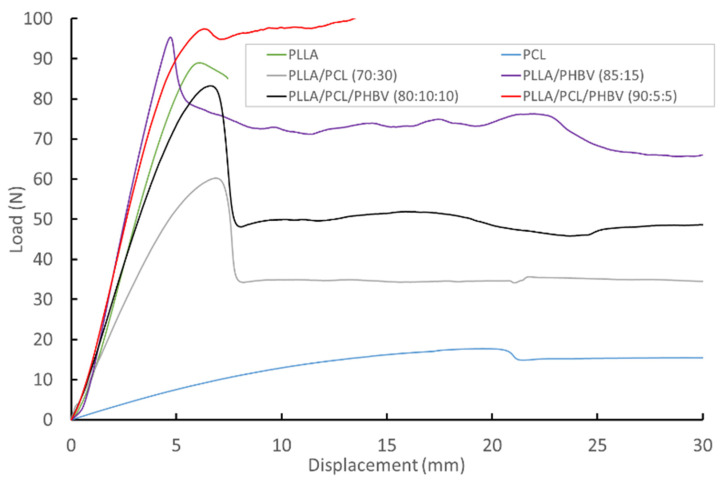
Representative load–displacement responses from filament tensile testing.

The results of the tensile tests on extruded filaments are summarised in Figure 4 and Table 3. PCL, when tested as a single polymer, displays the anticipated low modulus and high elongation behaviour. In the binary blend with PLLA, the inclusion of PCL reduces the average modulus and yield stress compared to PLLA alone but increases elongation at break. The addition of PHBV to PLLA slightly increases the average modulus and yield stress compared to PLLA alone, but also increases the elongation at break.

For the ternary blends, small amounts of PHBV increase the average modulus and yield stress, but as the amount of PCL increases, the effect of PCL on lowering the average modulus and yield stress also increases. For the ternary blends, the effect on the elongation at break is variable, but the average elongation at break achieved is always greater than that shown by PLLA alone.

**Figure 5:**
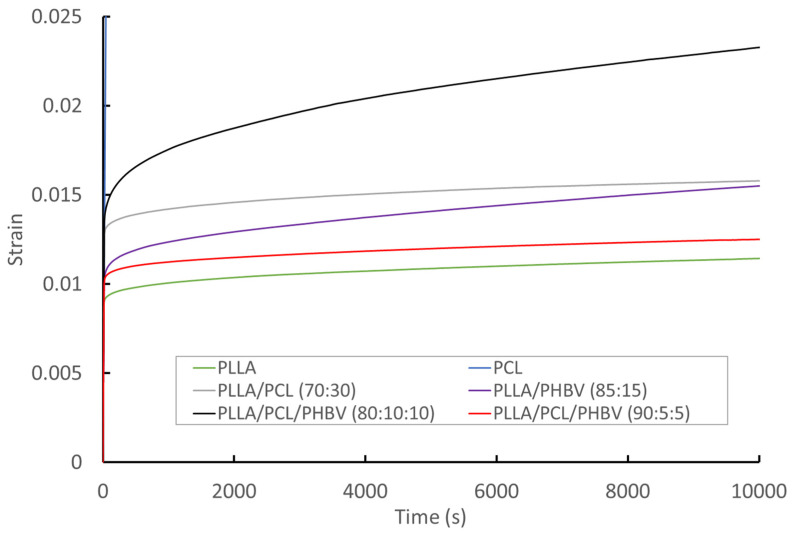
Representative time–strain curves for polymers and blend filaments under a 40 N constant load.

Figure 5 illustrates curves for material strain under constant load with respect to time for each of the polymers and blends at room temperature (22 °C), and Table 4 shows the steady state creep rate for the materials. For PCL, the strain rate is very high (the maximum test displacement of 500 mm was reached in 3000 s), so no creep rate was calculated for this material. For all other materials, the absolute strain remains below 3% over the course of the test. For the PLLA/PCL/PHBV 80:10:10 and PLLA/PHBV 85:15 blends, there is a sharper increase in strain over time in comparison to the other materials. PLLA is the polymer with the lowest deformation rate. The deformation rate increases when PCL and PHBV are added to the PLLA matrix, and the PLLA/PCL/PHBV (90/5/5) blend has the most comparable strain relationship with time to PLLA. 

For the binary blends, the viscosity of the materials (Table 4) broadly follows the trends indicated by the modulus and strength: blending PHBV with PLLA increases the viscosity, while blending with PCL reduces the viscosity. In the ternary blends, the effect of the PCL dominates, giving a reduced viscosity compared to PLLA alone. All of the materials show a reduction in viscosity with increasing frequency.

## 4. Discussion

It is useful to note that initially, across all of the test types, the single polymers show properties in line with those reported in the literature for those polymers. The main aim in creating the polymer blends was to enhance the properties of PLLA, and we demonstrate that it is possible to use relatively small amounts of PCL and PHBV to achieve this aim. Considering first the two-component blends, the addition of both PCL and PHBV produced polymer blends which were less brittle than PLLA alone processed in the same way. Previous studies have shown that PLLA can be made less brittle through the addition of PCL [12], as long as the PCL is well dispersed within the PLLA. The extrusion process used here seems to have achieved this dispersion. A number of studies have blended either PHB or PHBV with PLLA and observed improved toughness [24,25,26]. Blending PLLA with PCL reduces the crystallinity compared to PLLA processed alone using the same processing parameters, with this reflected in a reduced modulus and tensile strength. Blending PLLA with PHBV, however, increases the crystallinity compared to PLLA processed alone using the same processing parameters, with this reflected in an increased modulus and tensile strength, giving an almost ideal outcome of improved modulus, strength and elongation at break from blending. PHBV is considered to act as a nucleation agent for the PLLA [25], giving more crystallisation but smaller crystals, thus enhancing the mechanical properties across the board.

Interestingly, in the three-component PLLA/PCL/PHBV blends, the addition of PCL causes the crystallinity to fall to below that observed for PLLA alone, whilst maintaining the modulus and elongation at break at levels similar to those for PLLA alone, suggesting that whilst the PCL reduces the total amount of crystallinity, the refined crystal structure stimulated by the PHBV still enhances the mechanical properties. Overall, the mechanical properties of the PLLA/PHBV blend and the two PLLA/PCL/PHBV blends offer similar modulus and strength to that of PLLA alone, whilst improving the elongation at break, although for the 90/5/5 PLLA/PCL/PHBV blend, the improvement in elongation at break was limited. This combination of improved properties has not been attained in previously reported work on binary or ternary blends with PLLA.

When considering the creep behaviour of the polymers, the proportion of PCL in the ternary blends seemed to have a greater influence than it had on the initial mechanical properties. That the creep behaviour of the blends cannot be inferred from the trends seen in the initial mechanical properties is important to bear in mind when developing blended polymers for applications which involve the polymer being under load for an extended time period. The 80/10/10 blend had a higher initial strain under load, and a greater rate of strain accumulation than the PLLA, PLLA/PHBV or 90/5/5 ternary blend. We consider that the reduced crystallinity, reduced modulus and increased volume of PCL in the 80/10/10 blend made it easier for the polymer material to creep through increased localised yielding [40], which then led to an increase in the steady state creep rate.

The addition of PCL into the blends also has a greater impact on viscosity than on the static properties. Testing of molten material means that any benefits which are dependent on crystallinity are no longer applicable, but it is interesting to note that in the ternary blends, it is the influence of PCL which dominates over the PHBV, which in the PLLA/PHBV blend causes an increase in viscosity. In essence, it appears that the low-viscosity PCL acts effectively as a plasticiser for the PLLA and PHBV. This makes the blends which contain PCL easier to melt to create a component shape using conventional polymer processing techniques or, as we have filament raw material, the additive manufacturing technique of fused deposition modelling.

In evaluating materials for potential use in orthopaedic applications on the basis of their mechanical properties, we should first note that the materials described here are appropriate for cancellous bone applications (modulus in the range 10 MPa to 3 GPa, strength 0.1 to 30 MPa), as the mechanical properties do not approach those of cortical bone (modulus ~18 GPa; strength ~70 MPa) [41]. With that constraint, the combination of initial mechanical properties and creep behaviour mean that the PLLA/PHBV blend and the 90:5:5 PLLA/PCL/PHBV blend would be the most promising materials. The enhanced elongation at break of the PLLA/PHBV blend offers further value where greater elasticity is required. Future work will consider the biological properties of the materials, but the track record of the constituent materials in biomedical applications gives confidence that the materials will have biological properties appropriate for orthopaedic applications.

## 5. Conclusions

Blends of PLLA, PHBV and PCL were produced using twin screw extrusion and characterised using FTIR, DSC, tensile testing (including tensile creep) and rheometry. FTIR and DSC showed that all three materials were effectively processed and incorporated into the blends. The tensile, creep and viscosity data show that in small concentrations, PHBV alone (up to 25%) and PCL/PHBV together (up to 10% of each polymer) can modify the behaviour of PLLA in a positive way, offering improved modulus, strength and elongation at break depending on the concentrations of each polymer used. Within the constraints of the range of concentrations of the three polymers used here, the influence of PHBV outweighs that of PCL in terms of the effect on modulus and strength, whereas for creep behaviour and viscosity, the influence of the PCL outweighs that of PHBV. At a concentration of 5% PCL in the ternary blends, the creep rate exhibited by the polymer blend was identical to that of PLLA alone. Overall, we consider that blending PCL and PHBV through melt processing with PLLA offers an effective approach to the development of new polyester-based biomaterials with combinations of properties which cannot be provided by any of the materials individually, and which have not been demonstrated through previous approaches to blending.

## Figures and Tables

**Table 1:** Extrusion temperatures for each of the 5 temperature zones.

Polymer	Temperature (°C)				
	Z1	Z2	Z3	Z4	Die
PLLA and blended materials	125	240	240	220	180
PCL	95	120	100	100	90

**Table 2:** Thermal characteristics and crystallinity. *omr* indicates effect outside the measurement range; *nm* indicates effect not measurable. Results for blended materials are all post-extrusion. Δ*H_cc_* for samples which did not show a cold crystallinity peak assumed to be 0. PLLA and PHBV cold crystallisation peaks or melt peaks closely align, so a single value is reported. PCL melting peaks were distinct, and so the blends containing PCL values are reported separately for PCL and the other components of the blend.

Polymer	T_g_ (°C)	T_cc_ (°C)	T_m_ (°C)	$\Delta H_{m}$ (J/g)	$\Delta H_{cc}$ (J/g)	X_c_ (%)
PLLA (Pre-extrusion)	76	*nm*	190	74.4	~0	79.4
PLLA (Post-extrusion)	60	98	182	57.7	29.2	30.4
PCL (Pre-extrusion)	*omr*	*nm*	64	86.7	~0	62.4
PCL (Post-extrusion)	*omr*	*nm*	64	73.4	~0	53.0
PHBV (Pre-extrusion)	*omr*	*nm*	143/157	44.0	~0	30.0
PLLA/PCL (70:30)	68	*nm*	178	31.3 (PLLA); 5.9 (PCL)	~0	24.7
PLLA/PHBV (85:15)	59	83	178	53.6	20.2	42.5
PLLA/PCL/PHBV (80:10:10)	59	83	178	34.5 (PLLA and PHBV); 27.4 (PCL)	14.1 (PLLA and PHBV)	21.7
PLLA/PCL/PHBV (90:5:5)	54	90	180	37.5 (PLLA and PHBV); 2.3 (PCL)	9.0 (PLLA and PHBV)	26.5

**Table 3:** Mechanical properties for polymer filaments. Mean ± s.d. *n* = 3.

Material	Ratio (wt %)	E (GPa)	Yield Stress (MPa)	Elongation at Break (%)
PLLA	100	2.23 ± 0.14	34.4 ± 1.90	2.69 ± 0.31
PCL	100	0.27 ± 0.12	10.30 ± 3.10	No break
PLLA/PCL	70/30	1.40 ± 0.07	21.40 ± 2.94	No break
PLLA/PHBV	85/15	2.80 ± 0.49	42.94 ± 5.90	66 ± 17
PLLA/PCL/PHBV	90/5/5	2.65 ± 0.17	53.29 ± 4.62	2.94 ± 0.21
PLLA/PCL/PHBV	80/10/10	1.94 ± 0.11	36.80 ± 5.0	133 ± 10

**Table 4:** Average creep rate at room temperature under a 40 N constant load, and measured viscosity at 210 °C.

Material	Average Creep Rate (s^−1^) Mean ± s.d. *n* = 3	Viscosity at 1 Hz (Pa.s)	Viscosity at 600 Hz (Pa.s)
PLLA	1.31 ± 0.20	3148.3	941.2
PLLA/PCL (70/30)	1.35 ± 0.004	1245.9	391.4
PLLA/PHBV (85/15)	1.35 ± 0.034	2120.4	470.2
PLLA/PCL/PHBV (90/5/5)	1.33 ± 0.11	3482.3	960.7
PLLA/PCL/PHBV (80/10/10)	1.60 ± 0.10	2436.7	500.3

## Data Availability

Data supporting this publication are openly available at the following dois:10.5281/zenodo.7235496; 10.5281/zenodo.7235502; 10.5281/zenodo.7235530; 10.5281/zenodo.7235574.

## References

[1] Jin W., Chu P.K. (2018). Orthopedic implants. Encyclopedia of Biomedical Engineering 1–3.

[2] Moghaddam N.S., Andani M.T., Amerinatanzi A., Haberland C., Huff S., Miller M., Elahinia M., Dean D. (2016). Metals for bone implants: Safety, design, and efficacy. Biomanuf. Rev..

[3] Hofmann G. (1992). Biodegradable implants in orthopaedic surgery—A review on the state-of-the-art. Clin. Mater..

[4] Narayanan G., Vernekar V.N., Kuyinu E.L., Laurencin C.T. (2016). Poly (lactic acid)-based biomaterials for orthopaedic regenerative engineering. Adv. Drug Deliv. Rev..

[5] Urquijo J., Guerrica-Echevarria G., Eguiazábal J.I. (2015). Melt processed PLA/PCL blends: Effect of processing method on phase structure, morphology, and mechanical properties. J. Appl. Polym. Sci..

[6] Patrício T., Bártolo P. (2013). Thermal stability of PCL/PLA blends produced by physical blending process. Procedia Engineering.

[7] Mofokeng J.P., Luyt A.S. (2015). Dynamic mechanical properties of PLA/PHBV, PLA/PCL, PHBV/PCL blends and their nanocomposites with TiO2 as nanofiller. Thermochim. Acta.

[8] da Silva D., Kaduri M., Poley M., Adir O., Krinsky N., Shainsky-Roitman J., Schroeder A. (2018). Biocompatibility, biodegradation and excretion of polylactic acid (PLA) in medical implants and theranostic systems. Chem. Eng. J..

[9] Farah S., Anderson D.G., Langer R. (2016). Physical and mechanical properties of PLA, and their functions in widespread applications—A comprehensive review. Adv. Drug Deliv. Rev..

[10] Balani K., Verma V., Agarwal A., Narayan R. (2015). Physical, Thermal, and Mechanical Properties of Polymers. Biosurfaces.

[11] Wagner J.R., Mount E.M., Giles H.F. (2014). Polymer Structure. Extrusion.

[12] Fortelny I., Ujcic A., Fambri L., Slouf M. (2019). Phase Structure, Compatibility, and Toughness of PLA/PCL Blends: A Review. Front. Mater..

[13] Ulery B.D., Nair L.S., Laurencin C.T. (2011). Biomedical applications of biodegradable polymers. J. Polym. Sci. Part B Polym. Phys..

[14] Gunatillake P., Mayadunne R., Adhikari R. (2006). Recent developments in biodegradable synthetic polymers. Biotechnol. Annu. Rev..

[15] Munajad A., Subroto C. (2018). Fourier Transform Infrared (FTIR) Spectroscopy Analysis of Transformer Paper in Mineral Oil-Paper Composite Insulation under Accelerated Thermal Aging. Energies.

[16] Mohammadi M.S., Ahmed I., Muja N., Rudd C., Bureau M.N., Nazhat S.N. (2011). Effect of phosphate-based glass fibre surface properties on thermally produced poly(lactic acid) matrix composites. J. Mater. Sci. Mater. Med..

[17] Nair L.S., Laurencin C.T. (2007). Biodegradable polymers as biomaterials. Prog. Polym. Sci..

[18] Pouton C.W., Akhtar S. (1996). Biosynthetic polyhydroxyalkanoates and their potential in drug delivery. Adv. Drug Deliv. Rev..

[19] Doyle C., Tanner E., Bonfield W. (1991). In vitro and in vivo evaluation of polyhydroxybutyrate and of polyhydroxybutyrate reinforced with hydroxyapatite. Biomaterials.

[20] Gerard T., Budtova T. (2012). Morphology and molten-state rheology of polylactide and polyhydroxyalkanoate blends. Eur. Polym. J..

[21] Ostafinska A., Fortelny I., Nevoralova M., Hodan J., Kredatusova J., Slouf M. (2015). Synergistic effects in mechanical properties of PLA/PCL blends with optimized composition, processing, and morphology. RSC Adv..

[22] Todo M., Park S.-D., Takayama T., Arakawa K. (2007). Fracture micromechanisms of bioabsorbable PLLA/PCL polymer blends. Eng. Fract. Mech..

[23] Sultana N., Wang M. (2008). PHBV/PLLA-based composite scaffolds containing nano-sized hydroxyapatite particles for bone tissue engineering. J. Exp. Nanosci..

[24] Krishnan S., Pandey P., Mohanty S., Nayak S.K. (2015). Toughening of Polylactic Acid: An Overview of Research Progress. Polym. Technol. Eng..

[25] El-Hadi A.M. (2017). Increase the elongation at break of poly (lactic acid) composites for use in food packaging films. Sci. Rep..

[26] Ferreira B.M.P., Zavaglia C.A.C., Duek E.A.R. (2002). Films of PLLA/PHBV: Thermal, morphological, and mechanical characterization. J. Appl. Polym. Sci..

[27] Zhang M., Thomas N.L. (2011). Blending polylactic acid with polyhydroxybutyrate: The effect on thermal, mechanical, and biodegradation properties. Adv. Polym. Technol..

[28] Hedrick M.M., Wu F., Mohanty A.K., Misra M. (2020). Morphology and performance relationship studies on biodegradable ternary blends of poly(3-hydroxybutyrate-co-3-hydroxyvalerate), polylactic acid, and polypropylene carbonate. RSC Adv..

[29] Zhang K., Mohanty A.K., Misra M. (2012). Fully Biodegradable and Biorenewable Ternary Blends from Polylactide, Poly(3-hydroxybutyrate-co-hydroxyvalerate) and Poly(butylene succinate) with Balanced Properties. ACS Appl. Mater. Interfaces.

[30] Rao R.U., Venkatanarayana B., Suman K.N.S. (2019). Enhancement of Mechanical Properties of PLA/PCL (80/20) Blend by Reinforcing with MMT Nanoclay. Mater. Today Proc..

[31] Zhao H., Cui Z., Sun X., Turng L.-S., Peng X. (2013). Morphology and Properties of Injection Molded Solid and Microcellular Polylactic Acid/Polyhydroxybutyrate-Valerate (PLA/PHBV) Blends. Ind. Eng. Chem. Res..

[32] Javadi A., Pilla S., Gong S., Turng L.S. (2011). Biobased and Biodegradable PHBV-Based Polymer Blends and Biocomposites: Properties and Applications. Handbook of Bioplastics and Biocomposites Engineering Applications.

[33] Lajewski S., Mauch A., Geiger K., Bonten C. (2021). Rheological Characterization and Modeling of Thermally Unstable Poly(3-hydroxybutyrate-co-3-hydroxyvalerate) (PHBV). Polymers.

[34] Matta A., Rao R.U., Suman K., Rambabu V. (2014). Preparation and Characterization of Biodegradable PLA/PCL Polymeric Blends. Procedia Mater. Sci..

[35] Jia S., Yu D., Zhu Y., Wang Z., Chen L., Fu L. (2017). Morphology, Crystallization and Thermal Behaviors of PLA-Based Composites: Wonderful Effects of Hybrid GO/PEG via Dynamic Impregnating. Polymers.

[36] Lagazzo A., Moliner C., Bosio B., Botter R., Arato E. (2019). Evaluation of the Mechanical and Thermal Properties Decay of PHBV/Sisal and PLA/Sisal Biocomposites at Different Recycle Steps. Polymers.

[37] Chieng B.W., Ibrahim N.A., Yunus W.M.Z.W., Hussein M.Z. (2014). Poly(lactic acid)/poly(ethylene glycol) polymer nanocomposites: Effects of graphene nanoplatelets. Polymers.

[38] Jing N., Jiang X., Wang Q., Tang Y., Zhang P. (2014). Attenuated total reflectance/Fourier transform infrared (ATR/FTIR) mapping coupled with principal component analysis for the study of in vitro degradation of porous polylactide/hydroxyapatite composite material. Anal. Methods.

[39] Prasad B., Borgohain R., Mandal B. (2019). Advances in Bio-based Polymer Membranes for CO_2_ Separation. Advances in Sustainable Polymers.

[40] Bergström J.S., Hayman D. (2016). An Overview of Mechanical Properties and Material Modeling of Polylactide (PLA) for Medical Applications. Ann. Biomed. Eng..

[41] Morgan E.F., Unnikrisnan G.U., Hussein A.I. (2018). Bone Mechanical Properties in Healthy and Diseased States. Annu. Rev. Biomed. Eng..

